# Towards practical applications of quantum emitters in boron nitride

**DOI:** 10.1038/s41598-021-93802-8

**Published:** 2021-07-29

**Authors:** M. Koperski, K. Pakuła, K. Nogajewski, A. K. Dąbrowska, M. Tokarczyk, T. Pelini, J. Binder, T. Fąs, J. Suffczyński, R. Stępniewski, A. Wysmołek, M. Potemski

**Affiliations:** 1 Department of Materials Science and Engineering, National University of Singapore, Singapore, 117575 Singapore; 2 Faculty of Physics, University of Warsaw, Pasteura 5, 02-093 Warsaw, Poland; 3 Laboratoire National des Champs Magnétiques Intenses, CNRS-UGA-UPS-INSA-EMFL, Grenoble, France

**Keywords:** Single photons and quantum effects, Fluorescence spectroscopy, Confocal microscopy, Two-dimensional materials, Quantum optics, Design, synthesis and processing, Synthesis and processing

## Abstract

We demonstrate quantum emission capabilities from boron nitride structures which are relevant for practical applications and can be seamlessly integrated into a variety of heterostructures and devices. First, the optical properties of polycrystalline BN films grown by metalorganic vapour-phase epitaxy are inspected. We observe that these specimens display an antibunching in the second-order correlation functions, if the broadband background luminescence is properly controlled. Furthermore, the feasibility to use flexible and transparent substrates to support hBN crystals that host quantum emitters is explored. We characterise hBN powders deposited onto polydimethylsiloxane films, which display quantum emission characteristics in ambient environmental conditions.

## Introduction

One of the domains in photonics, constantly growing in relevance for contemporary and future communication technologies, is the generation of light that displays quantum qualities. The ability to procure, with high probability, an individual photon creates an opportunity to manipulate a state of a fundamental quantum system for the benefit of secure information carriage at the highest attainable speed. At present, various quantum emitters are investigated and considered for possible applications. The family of well-established quantum light sources, such as isolated semiconductor quantum dots^1,2^, single molecules^3,4^ and individual crystal defects^5,6^, has recently incorporated the emitting centres in thin layers of 2D materials^7–11^. The marriage of 2D world with quantum emitters is particularly appealing from the point of view of practical aspects. With constant advancements of fabrication procedures, it is feasible to design and create heterostructures which combine materials exhibiting a variety of optical and electrical properties. The selection of functionalities offered by thin films of 2D materials includes now generation of single photons.

Among 2D materials that host quantum emitters, hexagonal boron nitride (hBN) appears as an especially promising candidate which could fulfil many requirements for a practically useful single photon source. The emergent defect-related emitters give rise to photoluminescence in form of spectrally narrow lines^11–20^, which cover an exceptionally broad energy range, from near-infrared to ultraviolet optical regions. Such deep-level centers can constitute pure quantum systems, unperturbed by hybridization effects with fundamental electronic bands. The second-order photon correlation functions obtained for individual emission resonances reveal a good quality antibunching demonstrating quantum emission capabilities in cryogenic conditions and at room temperature. The samples investigated so far have been based mostly on commercially available hBN powders or thin hBN films mechanically exfoliated from bulk crystals^13^ or grown via deposition^21^ or epitaxy^22,23^ techniques. Most of these efforts are tailored towards achieving single crystal materials that host controllable defects. The inspection of the optical response of the emergent quantum emitters in a well-defined crystallographic environment is essential for achieving fundamental understanding of the optoelectronic properties originating from fundamental sub-bands and defect-induced midgap levels^24^. However, ample modern technologies rely on polycrystalline films and/or structures, hence the development of useful quantum emitters needs to take such technological challenges into account. Particularly, technological progress will require utilization of crystal growth techniques that are scalable and allow high-throughput fabrication of crystals that display quantum emission. Here, we explore the metalorganic vapour-phase epitaxy (MOVPE) techniques for fabricating hBN layers when we relax the condition of obtaining high-quality single crystals to accelerate the growth rate and efficiency while maintaining quantum emission capabilities.

From the point of view of optical characterisation of quantum light sources in hBN, the spectral properties of such emitters are quite well understood. Further progress in the field will likely depend on a possibility to tailor the parameters of the hBN samples and to realise novel ideas for incorporation of hBN quantum emitters into structures of practical importance. In this work we explore these concepts by (i) characterising the hBN samples grown by the method of MOVPE when using different process parameters and (ii) demonstrating that hBN powder emitters may act as efficient quantum emitters on flexible and transparent polydimethylsiloxane (PDMS) films in ambient atmosphere. The reported observations are relevant for the progress in development of technologically sound BN structures with the focus on quantum emission capabilities in a broad range of environmental conditions.

## Quantum emitters in MOVPE-grown polycrystalline hBN structures

**Figure 1:**
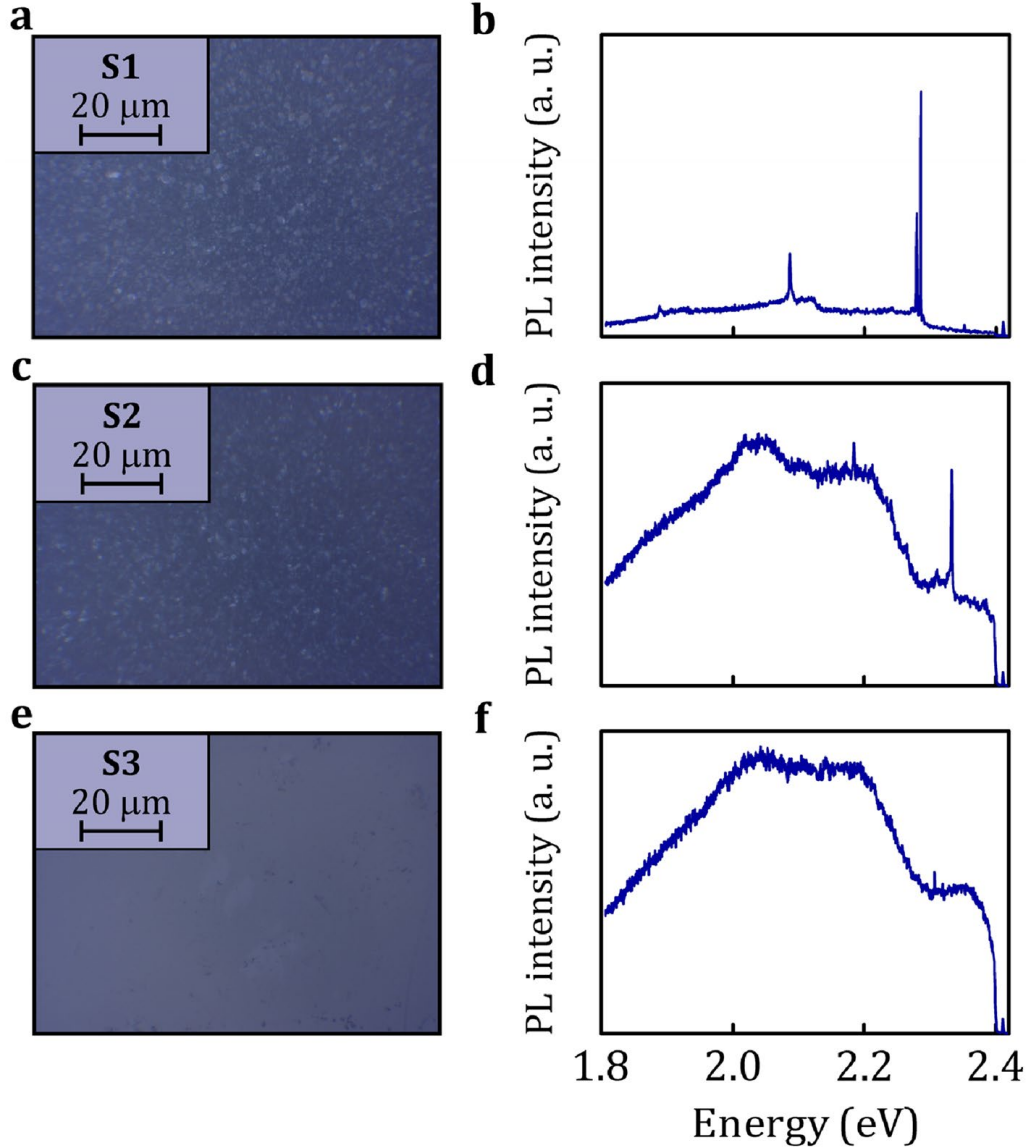
The optical images of Si/SiO_2_ substrates supporting transferred MOVPE hBN are confronted with typical μPL spectra taken on locations displaying emission from single defects for samples S1 (**a,b**), S2 (**c,d**) and S3 (**e,f**), respectively. The low temperature (5 K) μPL spectra were measured under 514 nm laser excitation with the power on the sample 300 μW for samples S1, S2 and 50 μW for sample S3. All studied samples host quantum emitters. However, for the specimen with strong broadband background it is infeasible to find suitable excitation conditions (e.g., resonant laser energy or linearly polarised excitation aligned with the intrinsic anisotropy axis of the emitter^13^), so that the narrow lines would be well pronounced (sample S3).

hBN polycrystalline films have been grown via MOVPE on sapphire supports using process parameters as listed in Table 1. For the optical study, the hBN structures were relocated onto Si/SiO_2_ substrates via pick-up transfer techniques utilising polydimethylsiloxane membranes. Generally, the photoluminescence (PL) spectra of our MOVPE hBN samples measured under conditions of the below bandgap excitation take form of broad emission bands. Such response of wide gap materials in visible spectral range may be related to ensembles of deeply bound defect states. Most importantly, the high-resolution μPL mapping reveals locations where individual narrow emission lines arise. Although all investigated samples reveal such narrow line emitters, the potential to isolate single PL resonances is limited by the apparent intensity of the broadband contribution. Representative spectra for samples S1–S3, showing signature of individual quantum emitters in our MOVPE hBN, are presented in Fig. 1. It is quite clear that the feasibility of an observation of individual defects in the optical spectra varies strongly across the investigated samples. Interestingly, the intensity of the background appears to be mostly related to the character of the crystallites that form the hBN layers, as may be seen in scanning electron microscopy images shown in Fig. S1 in Supplementary Information.

**Table 1:** The set of MOVPE growth parameters used to grow thick layers of polycrystalline hBN with crystals of various geometries.

	Carrier gas	Reactor pressure (mbar)	NH_3_ volume (cm^3^)	Estimated BN layer thickness (nm)
S1	H_2_	600	2000	800
S2	N_2_	600	1000	1250
S1	H_2_	100	100	550

See S. I., Fig. S1 for example SEM images.

**Figure 2:**
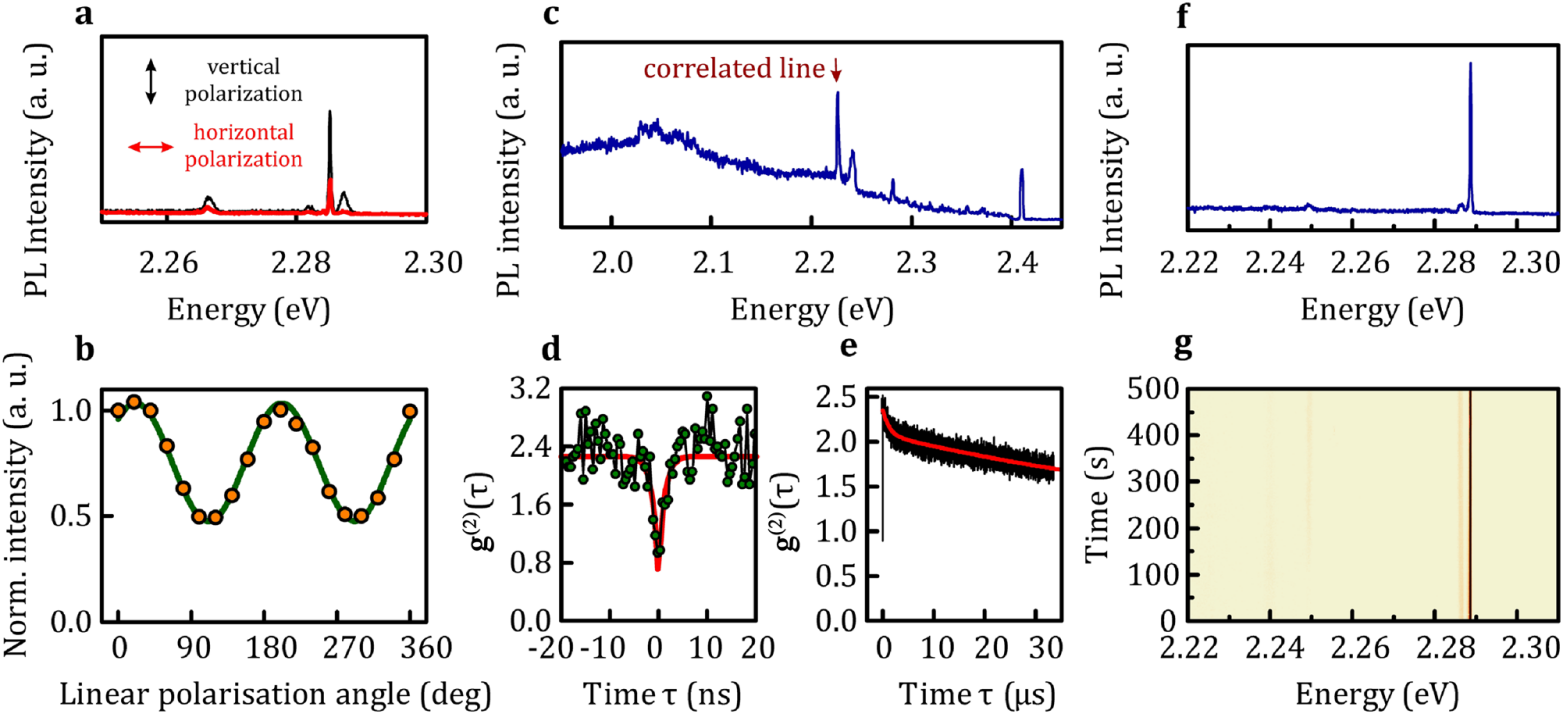
The low temperature (5 K) optical properties of the MOVPE-grown hBN. (**a**) The photoluminescence spectra resolved by the linear polarisation along two mutually perpendicular axes demonstrate that individual emission resonances display the high degree of linear polarisation (37 ± 1%). (**b**) A systematic dependence of the emission intensity of the most intense line in the spectrum may be reproduced by a squared sinusoidal function. Our individual BN emitters act as quantum emitters as presented for the resonance indicated in (**c**) in form of photon correlation function depicted (**d**) at shorter and (**e**) longer timescale. The stability of individual emission lines is very good for most emitters. This can be seen, e.g., for well-isolated resonances in (**f**), in a colour maps representing temporal evolution of the spectrum presented in (**g**).

A general inspection of the properties of quantum emitters in the MOVPE-grown hBN shows similar characteristics to those displayed by their counterparts in mechanically exfoliated layers and powders^11–15^. Representative results of the optical characterisation of the narrow line emitting centres in our samples are summarised in Fig. 2. Individual emitters exhibit a large degree of linear polarization of emitted light, which is indicative of an intrinsic anisotropy of the states partaking in the recombination processes. The angular dependence of the emission intensity is given as:

$$I\left( \alpha \right) = I_{A} \left( {\sin \left( {\alpha - \alpha _{0} } \right)} \right)^{2} + I_{0}$$

Here, $I_{A}$ is the amplitude of the angle $\left( \alpha \right)$-dependent oscillations, $\alpha _{0}$ is the phase shift with respect to an arbitrarily chosen axis of the linear polarizer and $I_{0}$ is the minimal intensity (displayed by the emitter when intrinsic polarisation axis is perpendicular to the axis of the polarizer). The luminescent centre, whose emission spectra and angle-dependent emission intensity is presented in Fig. 2a,b, displays a polarisation degree of:

$$P_{{deg}} = \frac{{I_{{max}} - I_{{min}} }}{{I_{{max}} + I_{{min}} }} = \frac{{I_{A} }}{{I_{A} + 2I_{0} }} = 37 \pm 1\% .$$

Characteristically for hBN quantum emitters, the second-order correlation function, obtained in Hanbury Brown and Twiss configuration, is dominated by a short time-scale antibunching^25^ in combination with a longer time-scale bunching. The shape of the bunching can be reproduced well by a bi-exponential decay function, so that the experimental data is well accounted for by the following formula:

$$g^{(2)} \left( \tau \right) = A_{1} e^{{{\raise0.7ex\hbox{${ - \left| \tau \right|}$} \!\mathord{\left/ {\vphantom {{ - \left| \tau \right|} {t_{1} }}}\right.\kern-\nulldelimiterspace} \!\lower0.7ex\hbox{${t_{1} }$}}}} + A_{2} e^{{{\raise0.7ex\hbox{${ - \left| \tau \right|}$} \!\mathord{\left/ {\vphantom {{ - \left| \tau \right|} {t_{2} }}}\right.\kern-\nulldelimiterspace} \!\lower0.7ex\hbox{${t_{2} }$}}}} - A_{3} e^{{{\raise0.7ex\hbox{${ - \left| \tau \right|}$} \!\mathord{\left/ {\vphantom {{ - \left| \tau \right|} {t_{3} }}}\right.\kern-\nulldelimiterspace} \!\lower0.7ex\hbox{${t_{3} }$}}}} + 1$$

with characteristic times $t_{1} = 1.3 \pm 0.1\,\upmu{\text{s}}$, $t_{2} = 77 \pm 1\,\upmu{\text{s}}$, $t_{3} = 1.2 \pm 0.3\,{\text{ns}}$ and bunching amplitudes $A_{1} = 1.1 \pm 0.1$, $A_{2} = 0.3 \pm 0.1$. The emergent anti-bunching is characterised by an amplitude that constitutes 70 ± 3% of the maximum number of counts.

**Figure 3:**
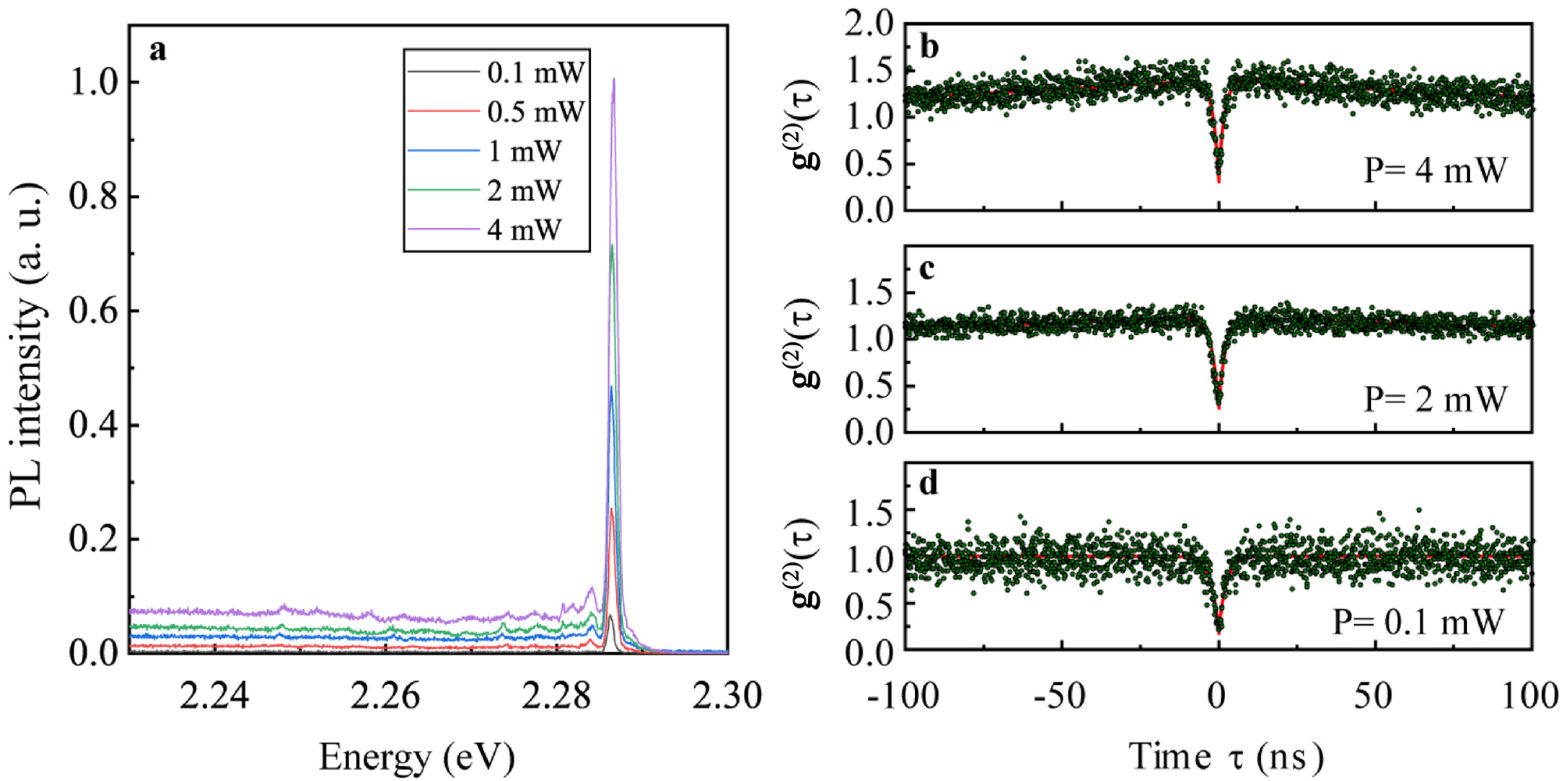
The power dependence of (**a**) the photoluminescence spectra obtained under 2.33 eV laser illumination in microscopic configuration at 1.8 K demonstrates the controllability of the broadband emission by the excitation conditions. Varying excitation power also impacts the bunching and antibunching amplitudes as seen in the g^(2)^(τ)﻿ functions for (**b**) 4 mW, (**c**) 2 mW and (**d**) 0.1 mW excitation power.

The parameters describing the g^(2)^(τ) function can also be altered by varying the excitation conditions. Figure 3 presents the dependence of the photoluminescence spectra and the correlation functions on the excitation power. Comparatively, this data demonstrates the decrease of the broadband background emission in the time-integrated spectra in conjunction with the decrease of the bunching amplitude for lower excitation powers. These two effects consequently decrease the value of  g^(2)^(0)﻿﻿. Particularly, in the regime of low excitation power (0.1 mW focused to a spot of about a micron diameter in our experiment), the broadband background contribution and the bunching effect are negligible. The parameters of the correlation function relevant for the quantum emission capabilities of this emitter are presented in Table 2.

**Table 2:** The fitting results for the experimental  g^(2)^(τ) functions from Fig. 3 are presented. For this data, A_2_ parameter was set to 0 as the bunching shape could be reproduced with a single time constant.

Laser power (mW)	Bunching amplitude (A_1_)	Antibunching amplitude (A_3_)	g^(2)^(0)
4	0.41 ± 0.01	1.10 ± 0.04	0.31 ± 0.05
2	0.24 ± 0.01	0.98 ± 0.03	0.26 ± 0.04
0.1	0	0.86 ± 0.04	0.14 ± 0.04

The other parameters were fitted with the exception of bunching amplitude for the g^(2)^(τ) function obtained under the condition of 0.1 mW laser excitation, for which the bunching amplitude is negligible.

## Quantum emitters in hBN powders on a flexible and transparent substrate

The appearance of quantum emitters in different forms of hBN, especially the polycrystalline structures, allows their seamless integration with a variety of modern technologies. Particularly important are optoelectronic structures and devices operating on transparent and/or flexible substrates^26–28^. Here, we demonstrate the quantum emission capabilities of hBN powders deposited onto polydimethylsiloxane (PDMS) films. Our micro-optical characterisation of such samples provides a proof of concept that quantum emitters in hBN may operate in ambient conditions on polymer films in a comparable fashion as on commonly used non-conductive crystal substrates.

**Figure 4:**
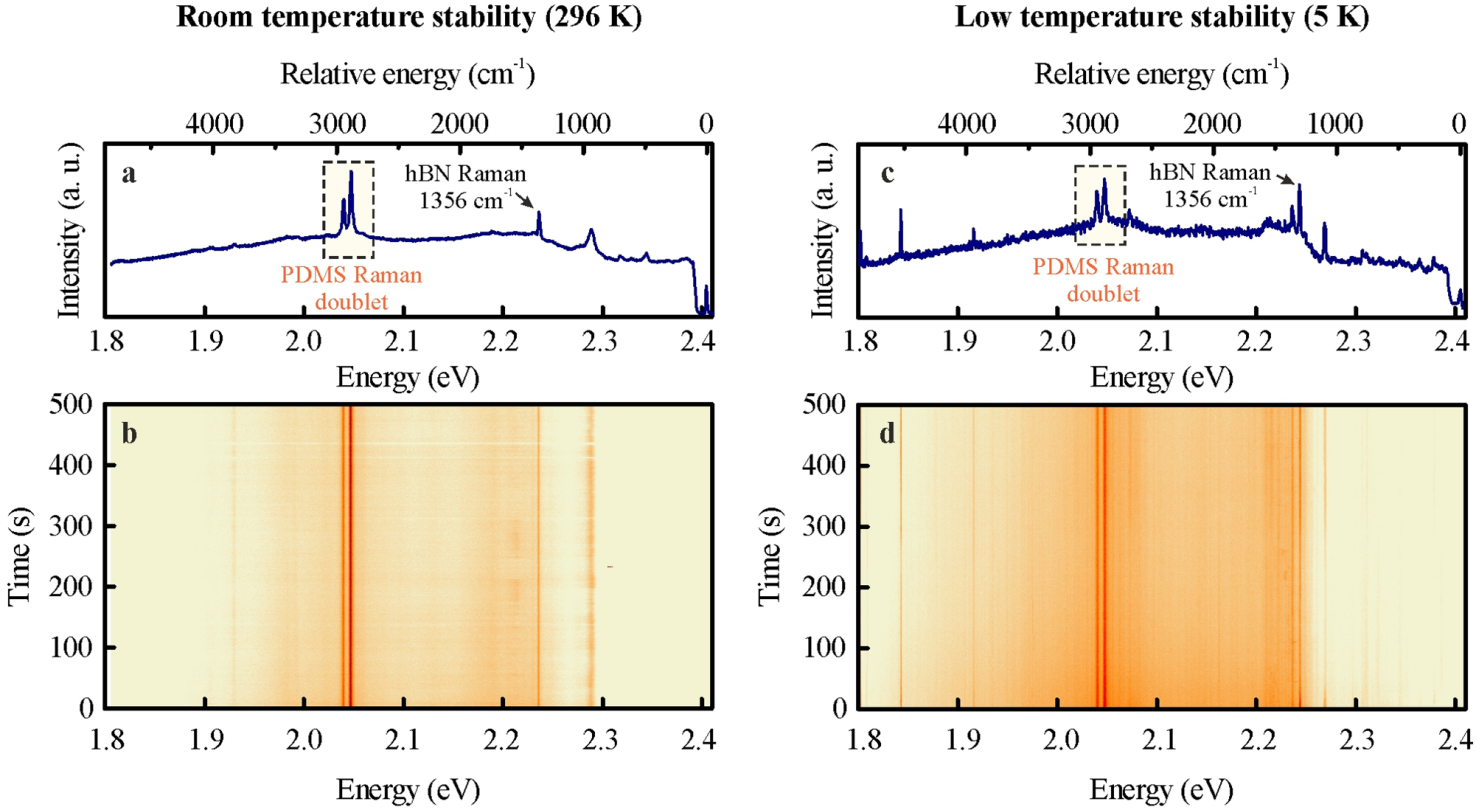
The optical response of hBN powder deposited onto PDMS films is presented at room temperature (**a**) and low temperature (**c**) conditions. The additional emission resonances accompany the PDMS Raman doublet and hBN optical phonon resonances. The hBN emission lines (e.g., that at 1.843 eV) display good stability at both cryogenic and ambient conditions as presented in the temporal evolution colour maps in (**b**,**d**).

In Fig. 4, we present a comparison between the room- and cryogenic-temperature optical response of the hBN powder deposited on a PDMS film under 2.410 eV (514.4 nm) laser excitation. In both cases, a doublet of Raman lines from PDMS polymer^29^ is conspicuous around 2920 cm^−1^ combined with a narrower hBN Raman resonance at 1356 cm^−1^. In the room temperature spectrum, presented in Fig. 4a, an additional broader line appears at 2.288 eV. At low temperature, the hBN emission resonances become significantly narrower, demonstrating that PDMS film may transfer heat efficiently even at cryogenic conditions, consequently allowing the hBN powder to thermalize.

In Fig. 4c, the most robust additional narrow resonances are seen at 1.843 eV and 2.269 eV. More examples of photoluminescence spectra, illustrating a broad spectral range wherein emission resonances from hBN powder may be found, are presented in S. I. in Fig. S3. A very good stability on the timescale of hundreds of seconds is observed for the hBN emission resonances in both ambient and low temperature experiments, as revealed by the temporal evolution of the optical spectra demonstrated in Fig. 4b,d.

**Figure 5:**
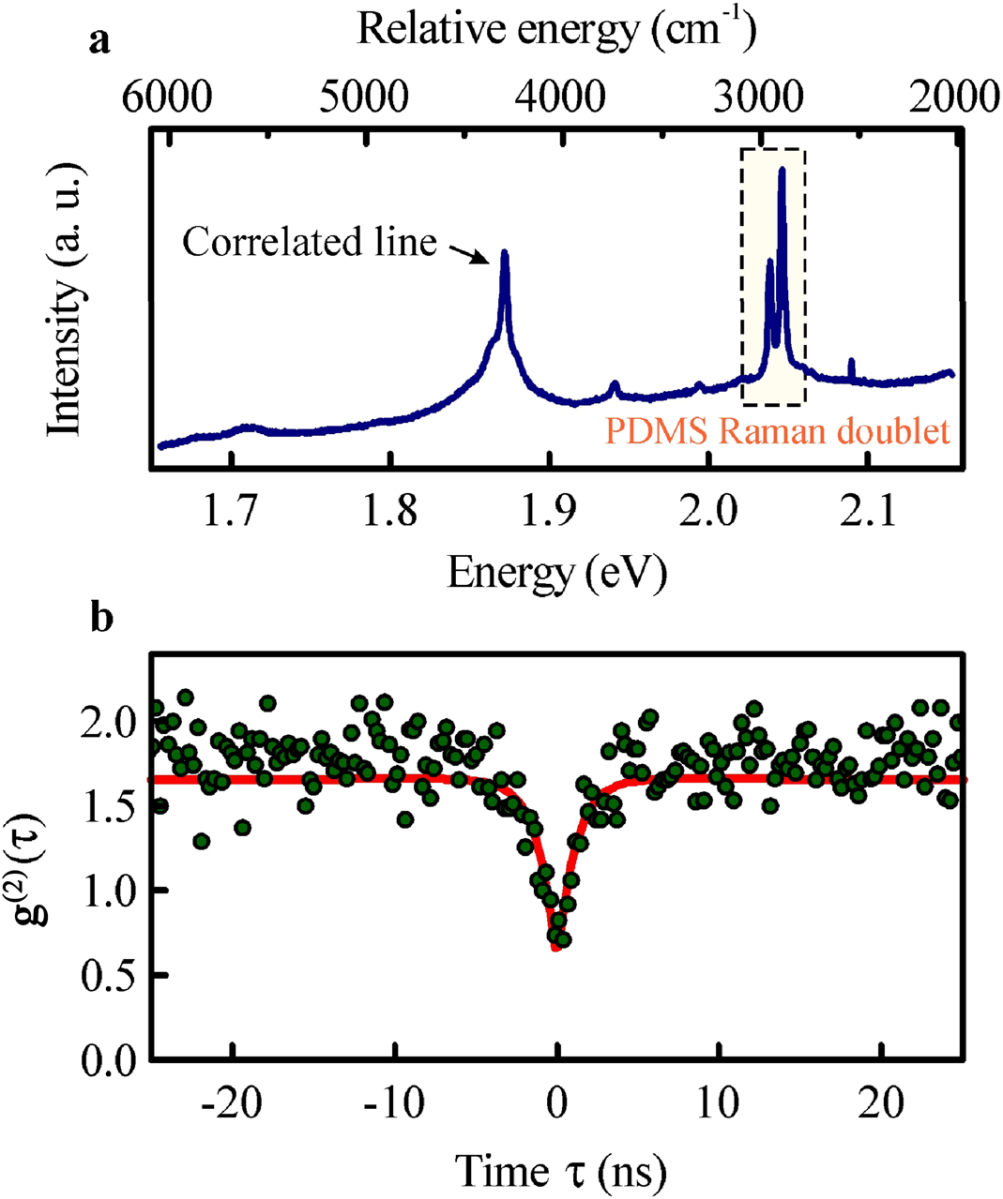
Room temperature quantum emission from hBN powder deposited onto PDMS film. The emission resonance used in second-order correlation function measurements is located at 1.872 eV as demonstrated in the optical spectrum (**a**) under 2.41 eV (514.4 nm) laser excitation. The $g^{(2)} \left( \tau \right)$ function displays an antibunching with the depth given by the amplitude that constitutes 62 ± 2% of the maximum number of counts.

The most relevant for practical aspects of a useful quantum emitter is the presence of an antibunching in photon correlation function in ambient conditions. It is indeed observable for the individual emission resonances displayed by hBN powder on PDMS samples, as we demonstrate in Fig. 5. Notably, the quantum emission from the hBN material is persevered under conditions suitable for practical operation of flexible optoelectronic devices. With PDMS being commonly used in exfoliation techniques^30^, a development of simple cavity systems^31^ or waveguides^32,33^, its combination with hBN powders creates an opportunity to utilize single photon sources in creative ways.

## Summary

We have explored technological aspects of utilising hBN quantum emitters as building blocks compatible with a broad range of modern technologies. Our MOVPE polycrystalline BN films display well-isolated and robust narrow-line resonances characterised by deep antibunching in a second-order correlation function. Such observation demonstrates that high-quality solid-state single quantum emitters do not necessarily require high-quality single crystals. Furthermore, there appears to be no stringent requirements for the substrates in order to achieve quantum emission from hBN structures in ambient conditions. As a proof of principle, we verify this notion by inspecting the optical response of quantum emitters in hBN on PDMS films, which may play a pivotal role in fabrication of 2D heterostructures and devices.

## Methods

### Crystal growth

The BN crystals were grown on two-inch sapphire wafers via MOVPE method using an Aixtron CCS 3 × 2″ system. Triethylborane (C_6_H_15_B, TEB) and ammonia (NH_3_) were used as the active agents acting as a source of boron and nitrogen, respectively. The growth temperature was controlled in situ by ARGUS Thermal Mapping System and for all samples was set to 1050 °C. The differences between the studied samples arise from the variation of growth parameters: carrier gas (nitrogen or hydrogen), reactor pressure and the volume of ammonia. The dependence of the properties and surface morphology on growth parameters has been further described in Ref. ^34^**.** All samples were grown in the conditions of ammonia insufficiency which promotes not only formation of nitrogen vacancies but also carbon incorporation into the boron nitride structure. Such growth strategies may open new pathways for controllable formation of specific luminescent centres. Relatively large amount of TEB results in a very effective material synthesis. Dense nucleation on the sapphire surface and appearance of defects during the growth rule out formation of epitaxial, continuous layer well aligned with the sapphire substrate and for this reason obtained boron nitride has a polycrystalline form.

### Sample preparation

The MOVPE-grown BN layers were transferred from the initial sapphire substrates to commonly used in optical spectroscopy Si/SiO_2_ substrates, allowing better visualisation of the BN layered via optical contrast methods. PDMS-based pick-up transfer technique was used to relocate the boron nitride crystals between various supports.

### Optical spectroscopy

The optical spectra were measured in a standard back-scattering geometry under a single-frequency 514.4 nm (2.410 eV) laser excitation. The laser beam was focused on the surface of the sample down to a spot of about 1 μm diameter with a long working distance 50 × magnification objective. The signal from the sample was spectrally resolved with a 500 mm spectrometer and detected by a charge-coupled device camera, producing an optical spectrum which consists of a combination of Raman and photoluminescence resonances. A flow helium cryostat equipped with a cold finger was used to cool the samples down to 5 K temperature. The optical spectra resolved by the linear polarisation were measured with a λ/2 waveplate followed by a linear polarised located in the detection path of the optical setup.

The photon correlation measurements were performed in a Hanbury Brown and Twiss configuration with the continuous wave 514.4 nm laser acting as an excitation source. Two avalanche photodiodes with a response time of 200 ps were used to detect the single photons and generate electrical signals. The delay between the electrical pulses was measured with a specialised correlation card module.

## Supplementary Information

Supplementary Information.
