# Supplementary material for: Towards practical applications of quantum emitters in boron nitride
Source: Sci Rep. 2021 Jul 29;11:15506. doi: 10.1038/s41598-021-93802-8 (PMC8322094; doi:10.1038/s41598-021-93802-8)
Supplement: Supplementary file 1 — Supplementary Information. [file 41598_2021_93802_MOESM1_ESM.pdf]

Supplementary Information (S. I.) for **Towards practical applications of quantum emitters in boron nitride**

M. Koperski[1], K. Pakuła[2], K. Nogajewski[2], A. K. Dąbrowska[2], M. Tokarczyk[2], T. Pelini[3], J. Binder[2], T. Fąs[2], J. Suffczyński[2], R. Stępniewski[2], A. Wysmołek[2], and M. Potemski[2,3]

[1]Department of Materials Science and Engineering, National University of Singapore, 117575, Singapore
[2]Faculty of Physics, University of Warsaw, Pasteura 5, 02-093 Warsaw, Poland
[3]Laboratoire National des Champs Magnétiques Intenses, CNRS-UGA-UPS-INSA-EMFL, Grenoble, France

1. Scanning electron microscopy (SEM) characterisation of the metal-organic vapour deposition (MOVPE)-grown hexagonal boron nitride (hBN) films.

The morphology of our MOVPE-grown films is starkly different for the three samples grown using various process parameters: hydrogen (H_2) or nitrogen (N_2) carrier gases as well as different values of reactor pressure or volume of ammonia (NH_3), as presented in the main text in **Table 1**. All the hBN layers are polycrystalline, however the size and shape of grains differ significantly across the three presented specimens. This is demonstrated by the SEM images shown in **Fig. S1**. Sample S1 is clearly granular with small grains evenly distributed over the surface of the substrate. Sample S2 is more disordered and it includes crystallites of various shapes including highly anisotropic geometries in form of thin needles. Sample S3 consists of a conglomerate of flakes aligned at various angles with respect to the surface of the sample, including parallel and perpendicular alignment.

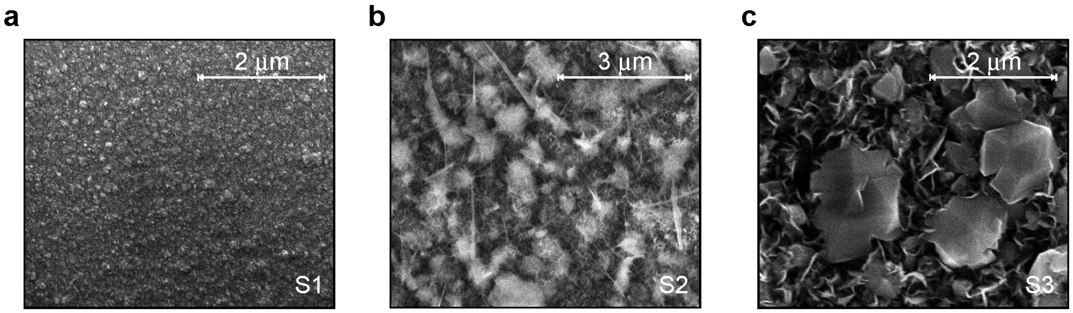

Figure S1. SEM images are presented for samples S1 **(a)**, S2 **(b)** and S3 **(c)** with MOVPE-grown BN films. In the SEM experiments, the focused beam of electrons was accelerated by high voltage of 15 kV for samples S1 and S3 and 10 kV for sample S2. The transmitted beam of electrons resulted in a probe current of 86 pA for all samples.

2. Optical response of the MOVPE-grown BN films.

The comparative analysis of the photoluminescence (PL) spectra displayed by samples S1, S2 and S3 allows to establish their suitability to observe, characterise and utilise single photon sources. A key parameter which determines such practical aspects in realistic samples is the ratio of the intensity of the spectrally narrow resonances to the intensity of the broadband background emission. Representative µPL spectra measured at different location from samples S1, S2 and S3 are presented in **Fig. S2**. These example spectra demonstrate that systematically the narrow line resonances are most conspicuous for sample S1. They are still observable in sample S2, however the presence of the background will limit the depth of the antibunching in the second-order correlation function, hence in realistic devices such hBN layers would not perform unambiguously as quantum emitters. In

sample S3, the narrow line resonances still exist, however the broadband background dominates the optical response of these films, hence it is practically unfeasible to perform a reliable characterisation of the quantum emitters in such hBN films.

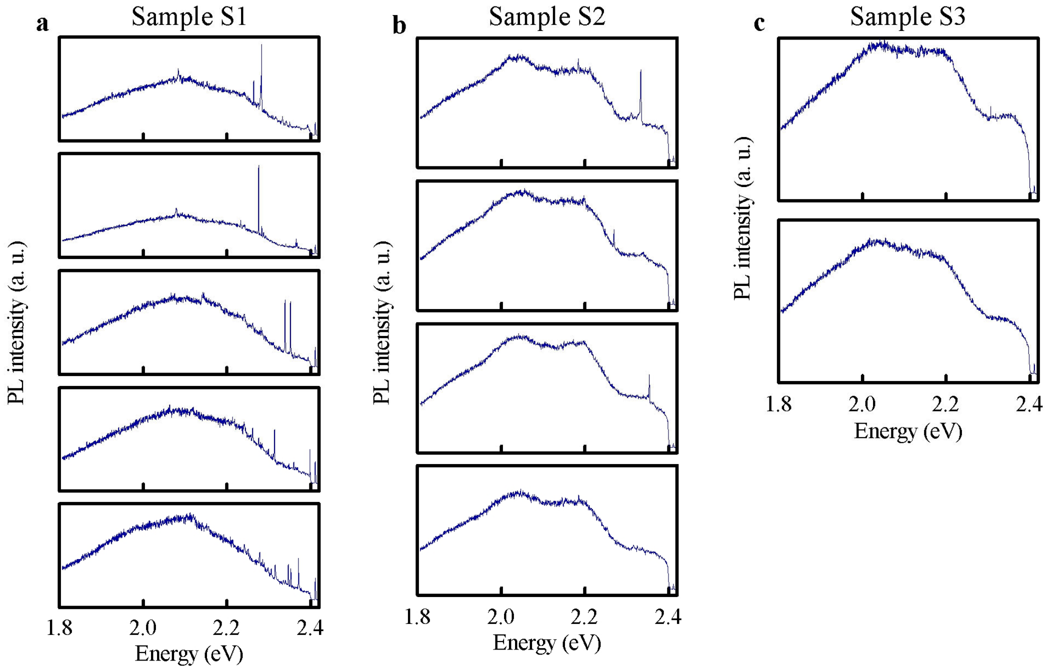

Figure S2. Representative low temperature (5 K) PL spectra are presented for samples S1 **(a)**, S2 **(b)** and S3 **(c)**. All the spectra were obtained under 514.4 nm (2.410 eV) laser excitation focused to a spot of about 1 μm^2 diameter. The power on the sample was set to 300 μW for samples S1 and S2 and 50 μW for sample S3. The shape and the intensity of the broadband background is rather homogenous in terms of intensity and spectral shape among all three samples. The narrow line resonances form unique pattern at specific spatial locations that can be found by scanning the surface of the samples.

3. Optical response of hBN powder deposited on polydimethylsiloxane (PDMS) films.

The hBN powder deposited on PDMS films shows diverse optical response. By scanning the surface of such films with a focused laser beam, one can identify locations that under the cryogenic conditions and at room temperature raise emission in form of spectrally narrow lines. Inspecting the representative PL spectra taken at different locations (see **Fig. S3**), we conclude that the spectral range where such resonances appear spans at least 0.5 eV. However, it is important to note this value is limited by our experimental capabilities in terms of the energy of excitation and the range of efficient detection. The resonances, although not very conspicuous, allow the measurements of the second-order photon correlation function, displaying photon antibunching even at room temperature as discussed in the main text.

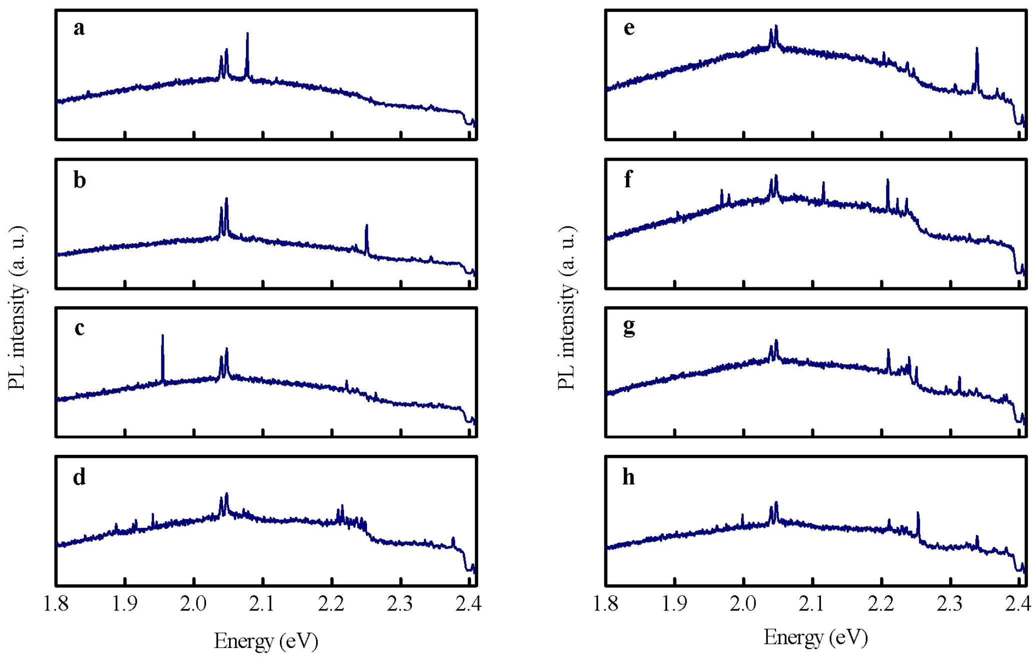

Figure S3. Representative PL spectra demonstrate the low temperature (5 K) emission of hBN powder at various locations **(a-h)** of a PDMS film attached to a cold finger in a helium flow cryostat. All spectra were obtained under 514.4 nm (2.410 eV) laser excitation focused to a spot of about 1 μm^2 diameter. The power on the sample was set to 200 μW.
